# Antioxidant Mechanisms of the Oligopeptides (FWKVV and FMPLH) from Muscle Hydrolysate of Miiuy Croaker against Oxidative Damage of HUVECs

**DOI:** 10.1155/2021/9987844

**Published:** 2021-08-22

**Authors:** Yue-Zhen Wang, Yu-Mei Wang, Xin Pan, Chang-Feng Chi, Bin Wang

**Affiliations:** ^1^Zhejiang Provincial Engineering Technology Research Center of Marine Biomedical Products, School of Food and Pharmacy, Zhejiang Ocean University, Zhoushan 316022, China; ^2^National and Provincial Joint Laboratory of Exploration and Utilization of Marine Aquatic Genetic Resources, National Engineering Research Center of Marine Facilities Aquaculture, School of Marine Science and Technology, Zhejiang Ocean University, Zhoushan 316022, China

## Abstract

In this work, the antioxidant mechanisms of bioactive oligopeptides (FWKVV and FMPLH) from protein hydrolysate of miiuy croaker muscle against H_2_O_2_-damaged human umbilical vein endothelial cells (HUVECs) were researched systemically. The finding demonstrated that the HUVEC viability treated with ten antioxidant peptides (M1 to M10) at 100.0 *μ*M for 24 h was not significantly affected compared with that of the normal group (*P* < 0.05). Furthermore, FWKVV and FMPLH at 100.0 *μ*M could very significantly enhance the viabilities (75.89 ± 1.79% and 70.03 ± 4.37%) of oxidative-damaged HUVECs by H_2_O_2_ compared with those of the model group (51.66 ± 2.48%) (*P* < 0.001). The results indicated that FWKVV and FMPLH played their protective functions through increasing the levels of antioxidant enzymes including superoxide dismutase (SOD) and glutathione peroxidase (GSH-Px) and decreasing the levels of reactive oxygen species (ROS), malondialdehyde (MDA), and nitric oxide (NO) in oxidative-damaged HUVECs in a dose-dependent manner. In addition, the comet assay revealed that FWKVV and FMPLH could dose-dependently protect deoxyribonucleic acid (DNA) from oxidative damage in the HUVEC model. These results suggested that antioxidant pentapeptides (FWKVV and FMPLH) could serve as potential antioxidant additives applied in the food products, pharmaceuticals, and health supplements.

## 1. Introduction

Bioactive peptides (BPs) produced from food resources are short amino acid sequences with 2 to 20 residues, which are inactive in the sequence of the original proteins [1–3]. Generally, BPs are liberated by protease hydrolysis during either food processing or gastrointestinal digestion in the body [4, 5]. Based on the types of amino acids, sequences, and spatial structures, BPs can effectively perform multiple functions, such as anticancer, antioxidant, angiotensin-converting enzyme (ACE) inhibition, immunomodulatory, antithrombotic hypocholesterolemic, antibacterial, hypotensive, and hypolipidemic activities [2, 6, 7], and their beneficial functions, especially on high nutritional value, health promotion, and chronic disease adjuvant treatment and prevention, are being increasingly recognized [4, 8].

Antioxidant peptides (APs) are one of the most popular types of BPs and have been isolated from animals, plants, and microorganism [9–11]. Liu et al. prepared the antioxidant peptide fraction from the processing of by-product of hazelnut, being composed of DWDPK, ETTL, ADGF, AGGF, AWDPE, and SGAF, which showed high antioxidant and protective ability against angiotensin (Ang) II-caused oxidant damage by upregulating the levels of superoxide dismutase (SOD) and heme oxygenase-1 (HO-1) and downregulating the level of xanthine oxidase-1 (XO-1) to control the generation of reactive oxygen species (ROS) in human umbilical vein endothelial cells (HUVECs) [12]. An antioxidant peptide YD1 from *Bacillus amyloliquefaciens* with average molecular weight (MW) of ∼1.0 kDa could strongly reduce the levels of ROS and nitric oxide (NO) and improve the levels of antioxidant enzymes by heightening the transcriptional and translational ability of NF-E2-related factor-2 (Nrf-2) in murine macrophages (RAW 264.7) [13]. EVSGPGLSPN from walnut showed high protection on PC12 cells against H_2_O_2_-caused neurotoxicity by controlling the nuclear factor kappa-B (NF-*κ*B)/caspase pathways and improving the activity of antioxidant enzymes, including glutathione peroxidase (GSH-Px), SOD, and catalase (CAT) [14].

At present, protein hydrolysates and BPs have been prepared from the processing of by-products of multifarious edible marine organisms, such as mollusks, fishes, and seaweeds, and many of them showed significant activities [2, 15–17]. Pan et al. found that four oligopeptides (VPR, IEPH, LEEEE, and IEEEQ) from protein hydrolysate of red stingray cartilages exhibited strong lipid peroxidation inhibition activity, Fe^2+^-chelating ability, reducing power, and radical scavenging activity [7]. Research by Oh et al. indicated that blue mussel-*α*-chymotrypsin hydrolysate (BMCH) showed high radical scavenging activity and inhibitory activity on Cu^2+^-mediated low-density lipoprotein (LDL) oxidation. Moreover, BMCH could improve the viability of HUVECs and lower ROS generation through downregulating gene expression of p53 and caspase-3, as well as decreasing the ratio of B-cell lymphoma 2- (bcl-2-) associated X (bax)/bcl-2 [18]. In addition, YPPAK, PIIVYWK, TTANIEDRR, and FSVVPSPK from blue mussel hydrolysate exhibited strong radical scavenging activity, and PIIVYWK and FSVVPSPK could activate of HO-1 gene expression to play their hepatoprotective effects [15, 19]. Chen and Hou reported that skin gelatin hydrolysate of Pacific cod could protect skin injury caused by ultraviolet (UV) radiation because the gelatin hydrolysate could restrain the decrease in endogenous antioxidases and hold back the expression of proinflammatory cytokines and NF-*κ*B [20]. Those studies confirm that APs could serve as antioxidant agents to inhibit the influences associated with oxidative stress in food and living organisms through enhancing the levels of endogenous antioxidant enzymes to prevent or repair the process of oxidation resulting from ROS [9, 15]. Furthermore, these APs showed excellent properties including being easy to absorb, having low MW, and being less toxic or having less side effect and become high-quality ingredients for healthy products and food processing/preservation industries [21, 22].

In our previous work, ten antioxidant peptides (M1 to M10) have been prepared from papain hydrolysate of miiuy croaker muscle and their amino acid sequences were determined to be YASVV (M1), NFWWP(M2), FWKVV (M3), TWKVV (M4), FMPLH (M5), YFLWP (M6), VIAPW (M7), WVWWW (M8), MWKVW (M9), and IRWWW (M10) [3]. In which, FWKVV (M3) and FMPLH (M5) could dramatically control lipid peroxidation and show powerful reducing power and radical scavenging ability. Therefore, the aim of this work was to evaluate the protective functions of FWKVV (M3) and FMPLH (M5) on HUVECs against hydrogen peroxide- (H_2_O_2_-) induced oxidative damage and illuminate their protective mechanism.

## 2. Material and Methods

### 2.1. Reagents

HUVECs were purchased from the Cell Bank of Type Culture Collection of the Chinese Academy of Sciences (Shanghai, China). Bicinchoninic acid (BCA), Dulbecco's modified Eagle's medium (DMEM), Hoechst 33342, ethidium bromide (EB), phosphate-buffered saline (PBS, pH 7.2), glycerol, bromophenol blue, acetylcysteine (NAC), L-glutamine, 3-(4,5-dimethylthiazol-2-y1)-2,5-diphenyltetrazolium bromide (MTT), and dimethyl sulfoxide (DMSO) were purchased from Sigma-Aldrich (Shanghai) Trading Co., Ltd. (China). The ten antioxidant peptides (M1-M10) were synthesized in China Peptides Co. (Suzhou, China), and the purity was more than 98% (*w*/*w*).

### 2.2. Culture and the Viability Assay of HUVECs

The culture method of HUVECs and the cell viability assay were carried out in accordance with the previous method of Cai et al. [23] and Lim et al. [24]. The MTT assay was replicated for three times and applied to determine the viability of HUVECs. In brief, the basal HUVECs were incubated in a 96-well plate for 24 h. After that, the basal HUVECs were cultured in the sample solutions with the concentration of 100 *μ*M. After 12 h, the wells were rinsed with PBS two times and the MTT was added to the wells with the final concentration of 0.5 mg/mL. After being incubated for 4 h, the active cells formed formazan crystals, which were added into 150 *μ*L of DMSO and well combined. Finally, the cell viability was determined according to the absorbance at 570 nm of the blank control group (*A*_control_) and the sample group (*A*_sample_) by the following formula:

(1) $$\begin{array}{c} Cell\;viability\;\left(\%\right)=\left(\frac{A_{sample}}{A_{control}}\right)\times 100\%. \end{array}$$

### 2.3. Protection of FWKVV (M3) and FMPLH (M5) on the Oxidative Damage Model of HUVECs

The oxidative damage model of HUVECs was established in accordance with the method described by Cai et al. [23]. After culturing for 24 h, the supernatant in HUVEC wells was aspirated. Subsequently, H_2_O_2_ was added and its final concentrations, respectively, reached 100, 200, 300, 400, and 500 mM. After 24 h, the HUVEC viability of each well was measured three times. In addition, the H_2_O_2_ concentration induced the cell viability; about 50% was selected to establish the oxidative damage model of HUVECs.

After establishing the oxidative damage model of HUVECs under the optimized H_2_O_2_ conditions, the peptide samples with the final concentrations of 10.0, 50.0, and 100.0 *μ*g/mL were added to the DMEM and well combined. After culturing for 24 h, the supernatant in HUVEC wells was wiped off. Afterwards, 100 *μ*L of the peptide samples at the given concentration was put in the protection groups. After 8 h, the peptide sample was cleared up and H_2_O_2_ at the given concentration was put in the model and peptide sample groups of HUVECs and treated for 24 h. 100 *μ*L of NAC (1.5 mM) was used in place of 100 *μ*L of peptide sample in the positive group.

### 2.4. Influences of FWKVV (M3) and FMPLH (M5) on the Morphology of HUVECs

The Hoechst 33342 staining assay was performed in accordance with the method described by Cai et al. [23]. After being treated by trypsinization, HUVECs were grown and incubated for 24 h in a 6-well plate. The supernatant in the 6-well plate was wiped off, and 300 *μ*L of NAC, FWKVV (M3), and FMPLH (M5) solutions was separately added into different 6-well plates. After incubating for 2 h, NAC, FWKVV (M3), and FMPLH (M5) were removed and 300 *μ*L of H_2_O_2_ was put in the plate separately. After incubating for 24 h, Hoechst 33342 was added into the plate Hoechst 33342 at a concentration of 8 mg/mL. After incubating for 30 min, Hoechst 33342 was cleared away from the plate and HUVECs were washed using serum-free DMEM three times. The fluorescence microscope of LSM710 was employed to observe the morphology of HUVECs, and 550 nm and 460 nm were designed as the excitation and emission wavelengths, respectively.

### 2.5. Influences of FWKVV (M3) and FMPLH (M5) on ROS, SOD, GSH-Px, NO, and MDA

The levels of ROS in blank control, model, and sample groups were measured in accordance with the method described by Cai et al. [23]. In addition, the levels of antioxidases (SOD and GSH-Px) and oxidation-related indicators (NO and MDA) were measured using assay kits in accordance with the manufacturer's protocols (Nanjing Jiancheng Bioengineering Institute Co., Ltd., China), and the BCA method was employed to determine the protein concentrations for normalizing the levels of antioxidases (SOD and GSH-Px). The level of ROS was indicated as % of blank control values. The levels of antioxidases (SOD and GSH-Px) were indicated as U/mg prot (units of enzymatic activity/mg protein).

### 2.6. Influence of FWKVV (M3) and FMPLH (M5) on Oxidative Damage to Deoxyribonucleic Acid (DNA) by H_2_O_2_

The protective ability of FWKVV (M3) and FMPLH (M5) on supercoiled pBR322 plasmid DNA against H_2_O_2_ damage was measured in accordance with the method described by Cai et al. [23] and Zhao et al. [25]. Glutathione (GSH) served as the positive control in the experiment.

The DNA comet assay was carried out in accordance with the method described by Cai et al. [23] and applied to analyze the protective ability of FWKVV (M3) and FMPLH (M5) on DNA damage in the oxidative damage model of HUVECs.

### 2.7. Statistical Analysis

All the assays were performed more than three times, and the resulting data were indicated as means ± standard deviation (SD). The mean value of each treatment was analyzed using the ANOVA test (SPSS Statistics 22.0 software). Duncan's multiple range tests were applied to analyze the significant differences of different groups (*P* < 0.05, *P* < 0.01, and *P* < 0.001).

## 3. Results and Discussion

### 3.1. Influence of APs (M1-M10) on the Viability of HUVEC

HUVECs are separated from the umbilical cord vein and play a major role as a model system for the analysis of the regulation of endothelial cells and the effect of antioxidants on endothelial cells and protection from oxidative damage by peroxide, measured by cell viability and its effect on apoptosis [5, 12].

**Figure 1:**
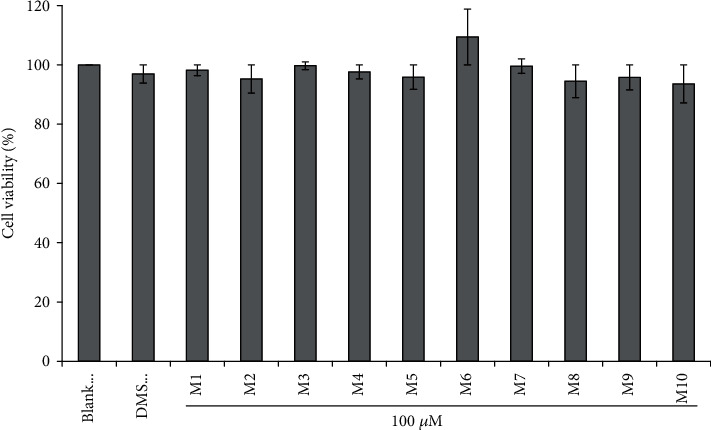
Influences of M1-M10 on the viability of HUVECs at the concentration of 100.0 *μ*M. The experiments were in triplicate (*n* = 3).

Figure 1 demonstrates the influence of APs (M1-M10) on cell viability. At the concentration of 100 *μ*M, the viability of HUVECs incubated with IRWWW (M10) for 24 h was 93.59 ± 6.41%, which was inferior to those of the blank control and other peptide groups. On the contrary, the viability of cells incubated with YFLWP (M6) was 109.39 ± 9.39%, which was superior to those of the blank control and other peptide groups. However, there was no significant difference between the control group and peptide-treated groups under the same conditions (*P* < 0.05). Cell viability is a determination of living or dead cells, based on a total cell sample. Viability analysis has a tremendous number of applications, especially calculating the effect of antitumor drug candidates on cancer cells and the cytotoxic activity of chemical compound on normal cells [26–28]. Therefore, ten isolated APs (M1-M10) from miiuy croaker revealed the possibility for exploiting nontumor healthy products because they had no significant influence on the conventional proliferation of HUVECs.

**Figure 2:**
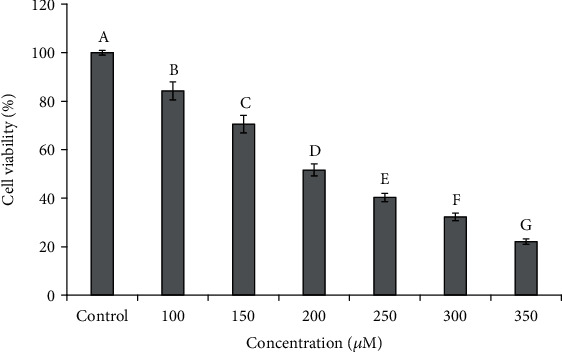
Influences of H_2_O_2_ concentrations (100.0 *μ*M to 350.0 *μ*M) on the viability of HUVECs. The experiments were in triplicate (*n* = 3). ^a-g^Values with the same letters indicate no significant difference (*P* > 0.05).

In the experiment, H_2_O_2_ was used to produce hydroxyl radicals (^∙^OH) and oxygen radicals, which further induce oxidative stress in cells [27]. Herein, the oxidative damage model built with H_2_O_2_ was usually applied to evaluate the antioxidant activity and investigate their molecular mechanisms concerned on the pathogenesis of ROS-caused oxidative injury.  Figure 2 shows that the viability of HUVECs decreased with the increase in H_2_O_2_ concentrations when the H_2_O_2_ concentrations ranged from 100 *μ*M to 350 *μ*M. In addition, it was found that the viability of HUVECs reduced to 51.66 ± 2.48% at the H_2_O_2_ concentration of 200 *μ*M, and there was significant difference with the other tested groups (*P* < 0.05). Then, 200 *μ*M was recognized as the optimal concentration of H_2_O_2_ for establishing the oxidative damage model of HUVECs.

### 3.2. Protective Effects of the APs (M1-M10) on the Oxidative Injury of HUVEC by H_2_O_2_

**Figure 3:**
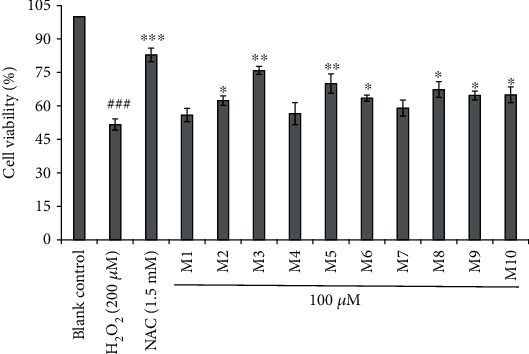
Influences of M1-M10 on the viability of oxidative-damaged HUVECs at the concentration of 100.0 *μ*M. ^###^*P* < 0.001 vs. the blank control group; ^∗^*P* < 0.05, ^∗∗^*P* < 0.01, and ^∗∗∗^*P* < 0.001 vs. the oxidative damage model group.

The protective ability of the 10 APs (M1-M10) on the oxidative injury model of HUVEC caused by H_2_O_2_ is presented in Figure 3. The results indicated that the cell viabilities of M1-M10-treated groups ranged from 55.90 ± 2.95% to 75.89 ± 1.79%, which were superior to that of the oxidative damage model group (51.66 ± 2.48%). However, the cell viabilities of M1-M10-treated groups were lower than that (82.90 ± 3.05%) of the positive control group (NAC). In addition, the HUVEC viability of the FWKVV- (M3) and FMPLH- (M5) treated group was 75.89 ± 1.79% and 70.03 ± 4.37% at the concentration of 100.0 *μ*M, which was very significantly (*P* < 0.001) superior to that of the oxidative damage model group (51.66 ± 2.48%); the NFWWP (M2), YFLWP (M6), WVWWW (M8), MWKVW (M9), and IRWWW (M10) increased the HUVEC viability to 62.36 ± 2.14%, 63.51 ± 1.34%, 67.34 ± 3.54%, 64.70 ± 1.94%, and 65.01 ± 3.52%, respectively, and the viabilities of those peptide-treated groups were significantly (*P* < 0.05) higher than that of the oxidative damage model group. Therefore, these results illustrated that FWKVV (M3) and FMPLH (M5) have the highest protective capability on HUVEC against oxidative damage induced by H_2_O_2_ among the 10 APs (M1-M10) when the concentration of APs (M1-M10) was 100.0 *μ*M.

**Figure 4:**
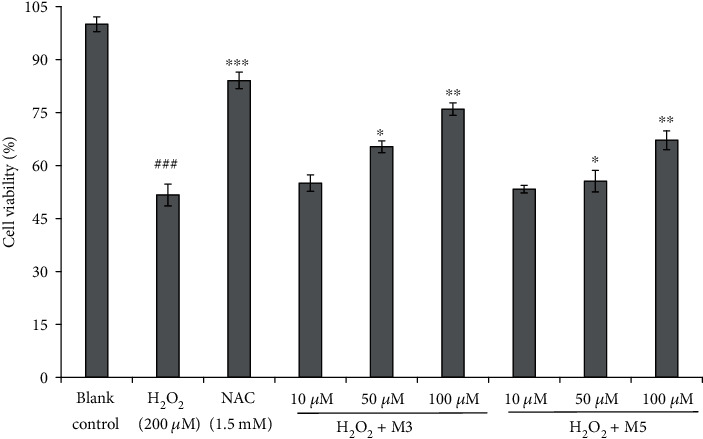
Influences of FWKVV (M3) and FMPLH (M5) on the viability of oxidative-damaged HUVECs at the concentrations of 10.0 *μ*M, 50.0 *μ*M, and 100.0 *μ*M. The experiments were in triplicate (*n* = 3). ^###^*P* < 0.001 vs. the blank control group; ^∗^*P* < 0.05, ^∗∗^*P* < 0.01, and ^∗∗∗^*P* < 0.001 vs. the oxidative damage model group.

The results in Figure 4 illustrated that the concentrations of FWKVV (M3) and FMPLH (M5) and their protective capability on the H_2_O_2_-induced HUVEC injury model showed a positive relationship. The HUVEC viability in the FWKVV- (M3) incubated group increased from 55.02 ± 2.35% to 75.89 ± 1.79% when its concentration was changed from 10.0 *μ*M and 100.0 *μ*M. Moreover, the HUVEC viability was very significantly (*P* < 0.01) and significantly (*P* < 0.05) higher than that of the oxidative damage model group at the concentrations of 50.0 *μ*M and 100.0 *μ*M, respectively. The HUVEC viability of the FMPLH- (M5) treated group showed similar tendency at the tested concentrations. However, there was no significant difference between FWKVV- (M3) and FMPLH- (M5) incubated groups and the oxidative damage model group at the concentration of 10.0 *μ*M (*P* > 0.05).

**Figure 5:**
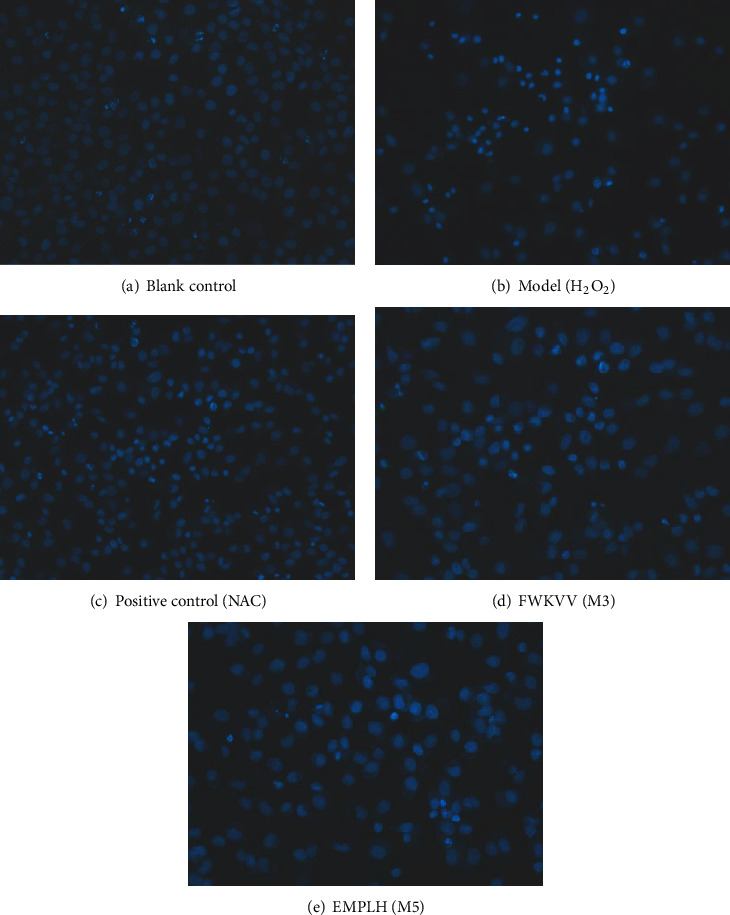
Influences of FWKVV (M3) and FMPLH (M5) on the apoptosis of oxidative-damaged HUVECs at the concentration of 100.0 *μ*M.

Images of the blank control (H_2_O), oxidative damage model (H_2_O_2_), FWKVV (M3), FMPLH (M5), and positive control (NAC) stained with Hoechst 33342 are presented in Figure 5. After incubation for 24 h, HUVECs were plump in shape, uniform in size, and presenting blue fluorescence in the blank control group (Figure 5(a)). However, HUVECs in the model group (Figure 5(b)) showed an apoptosis state because the number of HUVECs significantly reduced, the cells became smaller, and the fluorescence of most of the remaining adherent cells became bright. Compared with the model group, Figures 5(d) and 5(e) present that most of HUVECs in FWKVV (M3) and FMPLH (M5) groups adhered to the wall. In addition, only a handful of HUVECs were washed away and other small quantity of HUVECs that presented blue fluorescence brightened. However, the protective capacity of FWKVV (M3) and FMPLH (M5) on oxidative-damaged HUVECs induced by H_2_O_2_ was weaker than that of the positive control. The present results suggested that FWKVV (M3) and FMPLH (M5) displayed a significant protective capacity on the H_2_O_2_-damaged HUVECs, which agreed with the results found in Figure 2.

### 3.3. Effects of FWKVV (M3) and FMPLH (M5) on the ROS Levels in the Oxidative Damage Model of HUVECs

**Figure 6:**
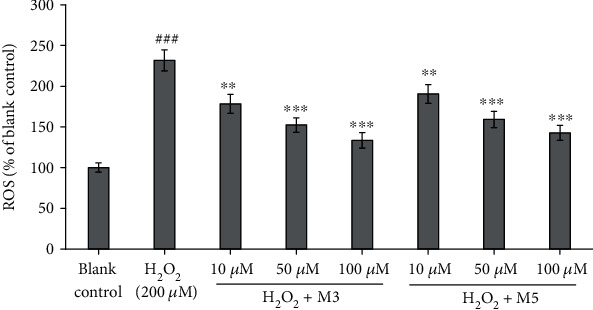
Influences of FWKVV (M3) and FMPLH (M5) on the levels of ROS in oxidative-damaged HUVECs at the concentrations of 10.0 *μ*M, 50.0 *μ*M, and 100.0 *μ*M. The experiments were in triplicate (*n* = 3). ^###^*P* < 0.001 vs. the blank control group; ^∗∗^*P* < 0.01 and ^∗∗∗^*P* < 0.001 vs. the oxidative damage model group.

The ROS levels in the blank control (H_2_O), oxidative damage model (H_2_O_2_), FWKVV (M3), FMPLH (M5), and positive control (NAC) groups are presented in Figure 6. The data demonstrated that there was an extremely (*P* < 0.001) significant difference between the blank control group and the oxidative damage model group (231.7 ± 12.8% of blank control). The finding proved that the induced oxidative damage caused by H_2_O_2_ resulted in a significant increase in ROS levels in the model group. In peptide groups, the intracellular ROS levels were significantly decreased by FWKVV (M3) and FMPLH (M5) pretreatment in a concentration-dependent manner. Moreover, FWKVV (M3) showed stronger activity of ROS scavenging than FMPLH (M5) did. At the concentrations of 10 *μ*M, 50 *μ*M, and 100 *μ*M, the intracellular ROS levels of the FWKVV- (M3) incubated group were 178.2 ± 11.7%, 152.3 ± 8.9%, and 133.4 ± 9.6% of the blank control group. The data indicated that FWKVV (M3) and FMPLH (M5) could play an important role in helping HUVECs from oxidative stress damage through decreasing the levels of ROS.

### 3.4. Effects of FWKVV (M3) and FMPLH (M5) on the Levels of Antioxidases (SOD and GSH-Px) and Oxidation-Related Indicators (NO and MDA) in the Oxidative Damage Model of HUVECs

**Figure 7:**
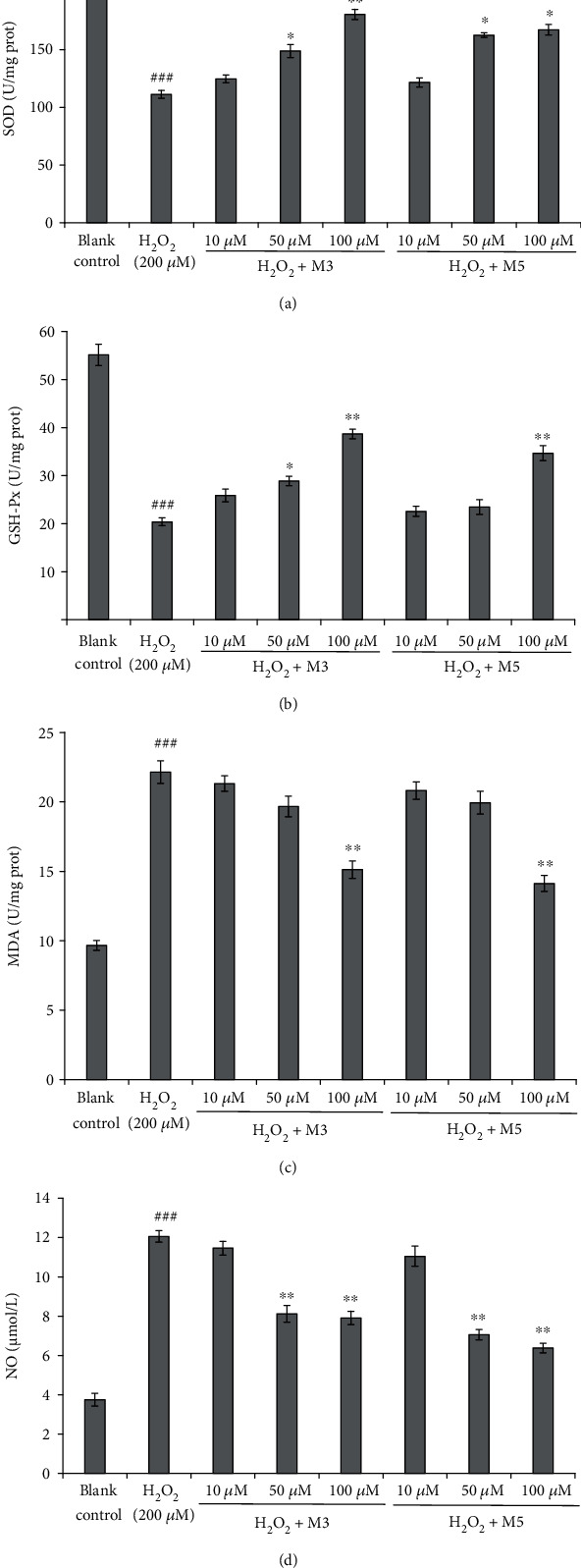
Influences of FWKVV (M3) and FMPLH (M5) on the levels of SOD (a), GSH-Px (b), MDA (c), and NO (d) in oxidative-damaged HUVECs at the concentrations of 10.0 *μ*M, 50.0 *μ*M, and 100.0 *μ*M. The experiments were in triplicate (*n* = 3). ^###^*P* < 0.001 vs. the blank control group; ^∗∗∗^*P* < 0.001, ^∗∗^*P* < 0.01, and ^∗^*P* < 0.05 vs. the oxidative damage model group.

The endogenous antioxidant enzymes, such as SOD, GSH-Px, CAT, and GSH-Rx, and nonenzymatic antioxidants form the body's endogenous defense mechanisms to protect vital biomolecules and ultimately body tissues against oxidative damage in cells through catalyzing reactions to neutralize ROS [29]. Under oxidative stress, uncontrolled generation of ROS will be beyond the normal processing capacity of this endogenous defense mechanism, and additional antioxidants are needed to help control oxidative stress damage [2, 30]. Hence, the influences of FWKVV (M3) and FMPLH (M5) on the levels of antioxidases (SOD and GSH-Px) and oxidation-related indicators (NO and MDA) in HUVECs were determined and are shown in Figure 7 for explaining their protective action on HUVECs against H_2_O_2_-induced oxidative damage.

Figures 7(a) and 7(b) show that the levels of antioxidases (SOD and GSH-Px) in the H_2_O_2_-induced HUVEC injury model were 111.35 ± 3.47 U/mg prot and 20.38 ± 0.82 U/mg prot, respectively, and the data were extremely significantly less than that of the normal HUVEC control (*P* < 0.001). Furthermore, Figures 7(a) and 7(b) indicate that there were positive correlations between the levels of antioxidases (SOD and GSH-Px) and the peptide (FWKVV (M3) and FMPLH (M5)) concentrations. The levels of SOD (148.94 ± 5.64%U/mg prot and 180.62 ± 4.38%U/mg prot) and GSH-Px (28.89 ± 0.96% and 38.67 ± 0.98%) of HUVECs incubated with FWKVV (M3) at the concentrations of 50.0 *μ*M and 100.0 *μ*M were very significantly (*P* < 0.01) and significantly(*P* < 0.05) higher than those of the oxidative damage model group. At the same concentration, the influence of FMPLH (M5) on the levels of antioxidases (SOD and GSH-Px) was slightly weaker than that of FWKVV (M3). The levels of SOD (162.64 ± 2.06 U/mg prot and 167.28 ± 4.57 U/mg prot) of HUVECs incubated with FMPLH (M5) at the concentrations of 50.0 *μ*M and 100.0 *μ*M were significantly (*P* < 0.05) and very significantly (*P* < 0.01) higher than those of the H_2_O_2_-damaged group. In addition, the levels of GSH-Px of HUVECs treated with FMPLH (M5) were 34.68 ± 1.55 U/mg prot at the concentration of 100.0 *μ*M, and the level was very significantly (*P* < 0.01) greater than that (20.38 ± 0.82 U/mg prot) of the oxidative damage model group. Therefore, the present finding indicated that FWKVV (M3) and FMPLH (M5), especially at the high concentration, had a significant influence on increasing the levels of antioxidases (SOD and GSH-Px) of HUVECs to deal with the damage of oxidative stress.

ROS generated under oxidative stress can damage the cell membrane and vital macromolecules, which causes the increase in MDA and NO. As shown in Figures 7(c) and 7(d), the contents of oxidation-related indicators including NO and MDA in the oxidative damage model group were significantly higher than those (MDA, 9.67 ± 0.35 nM/mg prot; NO, 3.75 ± 0.32 *μ*M/L) of the blank control group (*P* < 0.001). The results suggested that H_2_O_2_ causes oxidative stress in HUVECs at the concentration of 200 *μ*M and seriously damages the cell membranes. To our excitement, there were negative correlations between the levels of MDA and NO and the peptide (FWKVV (M3) and FMPLH (M5)) concentrations in Figures 7(c) and 7(d). The contents of MDA at the concentration of 100.0 *μ*M were 15.12 ± 0.62 nM/mg prot and 14.13 ± 0.58 nM/mg prot, respectively, and those data were significantly less than those of the oxidative damage model group (*P* < 0.01). The influences of FWKVV (M3) and FMPLH (M5) on the NO content in oxidative-damaged HUVECs were more significant than those on the MDA content. The contents of NO of HUVECs incubated with FWKVV (M3) and FMPLH (M5) at the concentration of 50.0 *μ*M were very significantly lower than those of the oxidative damage model group (*P* < 0.01). In FWKVV- (M3) and FMPLH- (M5) treated groups, the content decrease in MDA and NO in oxidative-damaged HUVECs further proved that FWKVV (M3) and FMPLH (M5) could increase the levels of antioxidant enzymes to scavenge ROS and alleviate its oxidized damage to cells.

Previous literatures indicated that some protein hydrolysates and BPs displayed intracellular antioxidant functions through controlling the levels and gene expression of antioxidant enzyme in cells and organisms [2]. Homayouni-Tabrizi et al. reported that YLEELHRLNAGY from camel milk protein hydrolysate could significantly increase the gene expression of SOD and CAT in treated HepG2 cells [31]. Antioxidant peptides derived from *Pinctada fucata* protein played a protective role in the ultraviolet-induced photoaging of mouse skins by significantly controlling the speed of lipid peroxidation and the reduction of the activity of SOD, GSH-Px, hydroxyproline, and CAT [32]. IYVVDLR and IYVFVR from the hydrolysate of soybean protein were able to protect Caco-2 cells against H_2_O_2_-induced oxidative damage *via* significantly downregulating intracellular ROS generation and lipid peroxidation, statistically upregulating total reduced glutathione (GSH) synthesis, enhancing activities of CAT and GSH-Px, and suppressing ROS-mediated inflammatory responses *via* inhibiting interleukin-8 secretion (*P* < 0.05) [33]. In an *in vivo* experiment, bovine hair hydrolysates (BHP) (with a major sequence of CERPTCCEHS) [34] and oyster meat hydrolysates by Alcalase (OMA) [35] showed a strong capability to decrease the MDA level and enhance the levels of endogenous cellular antioxidases, including SOD, CAT, and GSH-Px. Those findings in the experiment suggested that FWKVV (M3) and FMPLH (M5) had similar cytoprotective functions with those reported antioxidant peptides because they all could reinforce endogenous antioxidant defense systems to help cells against the H_2_O_2_ damage.

### 3.5. Protective Capacities of FWKVV (M3) and FMPLH (M5) on DNA

#### 3.5.1. Protective Capacities on Plasmid DNA

**Figure 8:**
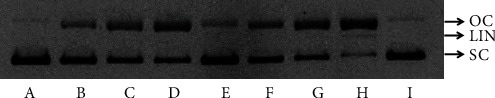
Protective capacities of FWKVV (M3) and FMPLH (M5) on plasmid pBR322 DNA against H_2_O_2_-induced oxidative damage. A: pBR322 DNA; B: pBR322 DNA+FeSO_4_+FWKVV (M3) (2.0 mg/mL)+H_2_O_2_; C: pBR322 DNA+FeSO_4_+FWKVV (M3) (1.0 mg/mL)+H_2_O_2_; D: pBR322 DNA+FeSO_4_+FWKVV (M3) (0.5 mg/mL)+H_2_O_2_; E: pBR322 DNA+FeSO_4_+FMPLH (M5) (2.0 mg/mL)+H_2_O_2_; F: pBR322 DNA+FeSO_4_+FMPLH (M5) (1.0 mg/mL)+H_2_O_2_; G: pBR322 DNA+FeSO_4_+FMPLH (M5) (0.5 mg/mL)+H_2_O_2_; H: pBR322 DNA+FeSO_4_+H_2_O_2_; and I: pBR322 DNA+FeSO_4_+GSH (2.0 mg/mL)+H_2_O_2_.

The superfluous ROS produced in oxidative stress can increase the level of the oxidative DNA damage, which is further implicated in the development of a variety of cancers including colon, breast, and prostate cancer [25, 36, 37]. Therefore, the protective effects of FWKVV (M3) and FMPLH (M5) on plasmid DNA (pBR322 DNA) against oxidative injury by H_2_O_2_ are displayed in Figure 8. It was found that the plasmid DNA (pBR322 DNA) is principally of the supercoiled (SC) form in normal circumstances (Figure 8(a)). A relaxed open circular (OC) form is produced when one of the phosphodiester chains of the plasmid DNA is cleaved when undergoing oxidative stress and further cut open surrounding the first breaking, which leads to generating the linear (LIN) double-stranded DNA molecules. The LIN and OC formations of DNA are characteristic of the double-strand and single-strand breaks, respectively [25].

In the experiment, the pBR322 DNA was cleaved by the hydroxyl radicals generated from the decomposition of H_2_O_2_ mediated by Fe^2+^, which changed the SC form into the OC form (Figure 8(h)). In addition, the trace LIN form of pBR322 DNA was discovered as shown in Figure 8(h), which suggested that a minute amount of the double strand of DNA was cut open by the superfluous hydroxyl radicals. As shown in Figures 8(b)–8(g), FWKVV (M3) and FMPLH (M5) positively affected the amount of the SC form of pBR322 DNA in a dose-dependent manner when their concentrations ranged from 0.5 mg/mL to 2.0 mg/mL. Correspondingly, the OC form of pBR322 DNA was gradually reduced with the increase in the concentrations of FWKVV (M3) and FMPLH (M5). However, the amount of the SC form of the plasmid DNA (pBR322 DNA) in the FWKVV (M3) and FMPLH (M5) group at the concentration of 2.0 mg/mL was less than that of the positive control (GSH) group (Figure 8(i)), which suggested that the protective effects on oxidative DNA damage of FWKVV (M3) and FMPLH (M5) were weaker than that of the positive control (GSH). Therefore, FWKVV (M3) and FMPLH (M5) might inhibit the chemical reaction of Fe^2+^ with H_2_O_2_ and/or directly clear away generated hydroxyl radicals through giving out the hydrogen atom or electron to play their defensive functions on the supercoiled plasmid DNA. This result agreed with our previous report that FWKVV (M3) and FMPLH (M5) had strong ferric reducing power and could effectively scavenge hydroxyl radical *in vitro* [3].

#### 3.5.2. Protective Capacities on DNA against H_2_O_2_-Induced Injury in the HUVEC Model

Due to the imbalance between the oxidants and antioxidants, redundant ROS is generated and then attacks DNA in cells, which further leads to adverse physiological and biochemical reactions. At present, several methods are applied to estimate the DNA damage in cells, and the comet assay is believed to be a fairly simple, sensitive, and versatile way in single cells to quantitatively and qualitatively evaluate the effects of DNA damage and DNA repair [37]. In the assay, an electrical current is applied to a slide featuring the cell in a gel. The broken strands of DNA will get away from their initial position towards the positive charge and leave a comet trail, which will be easily observable through fluorescence [38]. The comet trail can give out many quantitative data on the degree of DNA damage, which can serve to evaluate the protective effects of antioxidant compounds on DNA.

**Figure 9:**
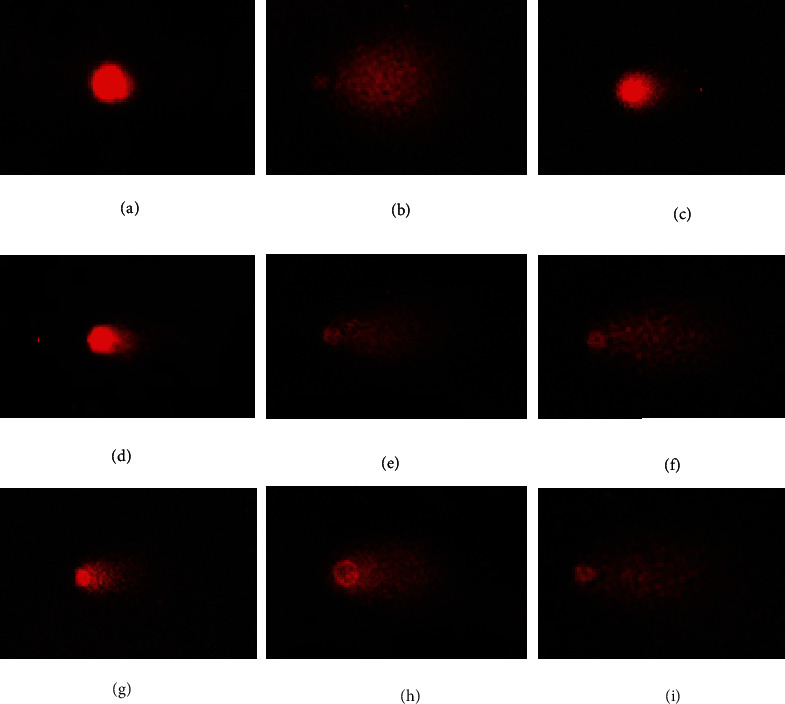
Protective capacities of FWKVV (M3) and FMPLH (M5) on DNA against H_2_O_2_-induced oxidative damage in HUVECs: (a) the blank control group; (b) the oxidative damage model group (H_2_O_2_, 200.0 *μ*M); (c) positive control (NAC) group; (d) H_2_O_2_+FWKVV (M3) (200.0 *μ*M); (e) H_2_O_2_+FWKVV (M3) (100.0 *μ*M); (f) H_2_O_2_+FWKVV (M3) (50.0 *μ*M); (g) H_2_O_2_+FMPLH (M5) (200.0 *μ*M); (h) H_2_O_2_+FMPLH (M5) (100.0 *μ*M); (i) H_2_O_2_+FMPLH (M5) (50.0 *μ*M).

In the blank control group, the comet with a bright head and almost without comet tail (Figure 9(a)) indicated that the DNA in HUVEC was in a normal state, but the comet with a long and large tail in the oxidative damage model group (Figure 9(b)) suggested that the DNA was seriously injured by H_2_O_2_ and the H_2_O_2_-induced oxidative damage model of HUVECs was successfully established. In comparison with the oxidative damage model group, the length and area of comet tails on oxidative-damaged HUVECs were step by step diminished with the increase in the concentrations of FWKVV (M3) and FMPLH (M5) from 50.0 *μ*M to 200.0 *μ*M (Figures 9(d)–9(i)). Even more interesting was that the length and area of comet tails in FWKVV- (M3) and FMPLH- (M5) incubated groups at the concentration of 200 *μ*M were approximately equal to the length and area of the NAC-treated group (Figure 9(c)).

The images in Figure 9 on the comet tail length or comet area could display visually the experimental results. Moreover, the comet assay had employed some indexes including torque class indicator (Olive tail moment, OTM), head DNA (HDNA), and tail DNA (TDNA) for being more conducive to accurate analysis of the results (Tables 1 and 2). These indexes demonstrated that the HDNA was extremely prominently lower than that of the blank control group (*P* < 0.001); the tail moment (TM), tail length (TL), OTM, and TDNA in the oxidative damage model group were extremely prominently higher than those in the blank control group (*P* < 0.001), and the comet length (CL) in the oxidative damage model group was significantly higher than that of the blank control group (*P* < 0.05). With the increase in the concentration of FWKVV (M3) and FMPLH (M5) from 50.0 *μ*M to 200.0 *μ*M, the TDNA, CL, TL, TM, and OTM decreased gradually, but the HDNA of the comet increased step by step. Furthermore, all the measured indicators of FWKVV (M3) and FMPLH (M5) groups at the concentrations of 100 and 200 *μ*M, except the CL of the FMPLH (M5) group at 100 *μ*M, showed extremely significant differences with those of the model groups (*P* < 0.001). At the concentration of 50 *μ*M, the HDNA, TL, and TM of FWKVV (M3) and FMPLH (M5) groups showed very significant (*P* < 0.01) differences with those of the model groups, respectively; the TDNA and OTM of FWKVV (M3) and FMPLH (M5) groups showed very significant differences with those of the model groups, respectively (*P* < 0.01); the OTM of FWKVV (M3) and FMPLH (M5) groups showed significant (*P* < 0.05) differences with that of the model groups, respectively, but the CL of FWKVV (M3) and FMPLH (M5) groups showed no significant differences with that of the oxidative damage model groups (*P* > 0.05). In consequence, the results of the comet assay demonstrated that FWKVV (M3) and FMPLH (M5) showed significantly protective capacity on DNA in the oxidative damage model groups induced by H_2_O_2_. In addition, FWKVV (M3) showed stronger protective effect on DNA in the H_2_O_2_-induced HUVEC injury model than FMPLH (M5) did at the same concentrations.

Attack of DNA by ROS produced under conditions of oxidative stress can induce strand breaks, DNA-DNA and DNA-protein crosslinking, and formation of at least 20 modified bases adducts, which play a key role in aging, cancer, arteriosclerosis, neurodegenerative diseases, and diabetes [39, 40]. The antioxidant hydrolysate and BPs are stage by stage accepted as food components applied in functional food and nutraceuticals to effectively adjust and control the oxidative injury to protect DNA, lipid, and protein in the human body [41–43]. Hoki frame protein hydrolysate (APHPH) could decrease t-butyl hydroperoxide-caused cytotoxicity on human embryonic lung fibroblasts and observably protect DNA against the free radical-induced injury [44]. Similarly, protein hydrolysates prepared from Nile tilapia [45] and shrimp shell [46] could effectively inhibit the DNA scission caused by H_2_O_2_ and peroxyl radical in a dose-dependent manner. YGDEY could protect HepG2 cells against alcohol-caused oxidative damage by controlling oxidative stress including reducing the degree of DNA damage. The antioxidant mechanism might be bound up with the Akt/NF-*κ*B/MAPK signal transduction pathways [47]. Sheih reported that VECYG (VG5) presented the protective ability on DNA against oxidative injury, so it further reinforced the capacity of the APs to protect hydroxyl radical-caused injury [48]. WAFAPA and MYPGLA prepared from the protein hydrolysate of the blue-spotted stingray were superior to carnosine in their capacity to control lipid oxidation caused by H_2_O_2_. In addition, WAFAPA and MYPGLA could help plasmid DNA against oxidative injury caused by Fenton's reagent [21]. In our previous report, FWKVV (M3) and FMPLH (M5) have been found to have strong lipid peroxidation inhibition, reducing power, and radical scavenging activities [3], and the current study finding demonstrated that FWKVV (M3) and FMPLH (M5) could weaken the oxidative injury of HUVECs caused by H_2_O_2_ through enhancing the contents of endogenous antioxidases (SOD and GSH-Px), lowering the contents of oxidation-related indicators (NO and MDA), and helping DNA from oxidative injury.

## 4. Discussion

Oxidative stress contributes to cell pathogenesis [49], ROS-mediated damage to DNA [39, 40], and an altered mitochondrial function [50]. The oxidative damage to DNA is considered a key factor in vascular disorder [51, 52]. Our results showed that the cell viability was significantly decreased, accompanied by DNA damage detected by the DNA comet assay and plasmid DNA assay after being cultured in 200 *μ*M H_2_O_2_ medium for 24 h, while H_2_O_2_-induced cytotoxicity and DNA damage were significantly inhibited when HUVECs were coincubated with FWKVV (M3) or FMPLH (M5). The study also found that the exposure of HUVECs to 200 *μ*M H_2_O_2_ for 24 h led to a dramatic increase in ROS production and induced apoptosis. However, treatment with M3 and M5 together with 200 *μ*M H_2_O_2_ markedly inhibited ROS formation and diminished DNA damage and cell apoptosis in HUVECs. These data suggest that M3 and M5 have ameliorative effects on H_2_O_2_-induced endothelial damage in HUVECs.

Excessive oxides deplete tissues of GSH and impair antioxidant defense systems in humans [53, 54]. How to increase the level of GSH becomes important. In this experiment, we observed that the levels of GSH in HUVECs had a marked increase in the groups with the treatment with M3 or M5. The results for the potential mechanism of the promoting production of GSH by M3 or M5 might contribute to a broader biological effect of a protective nature with regard to the general detoxification of environmental agents. No evidence was found that GSH was able to pass through cell membranes freely; thus, M3 or M5 must modulate GSH levels by stimulating cells to synthesize GSH intracellularly. The results indicate that M3 or M5 does protect cells from oxidative stress damage, and the protection includes its capacity to stimulate GSH synthesis.

Cells are in a stable state known as redox homeostasis under normal physiological conditions. Redox homeostasis is maintained by the balance between continuous ROS generation and several mechanisms involved in antioxidant activity [30, 55]. An overwhelming production of ROS leads to a prooxidant state also known as oxidative stress, which is a leading factor in the pathogenesis of vascular disorder complications [2, 56, 57]. NOX is a major contributor to ROS generation in endothelial cells [58]. Discrete compartmental redox signaling is demonstrated by Nrf2-dependent activation of ARE. Nrf2 is a redox-sensitive transcription factor that is activated by an oxidative signal in the cytoplasm. The Nrf2 pathway is regarded as the most important in the cell to protect against oxidative stress [40, 59]. Upon extracellular stimulation, the nucleus translocation of Nrf2 induces the transcription of several antioxidant genes, such as NADPH quinineoxidoreductase-1 (NQO-1), heme oxygenase 1 (HO-1), and SOD [60–62]. SOD and GSH-Px are two important antioxidant enzymes that remove toxic free radicals [29, 47]. Our study revealed that intracellular ROS were significantly increased when HUVECs were cultured in 200 *μ*M H_2_O_2_ media. Treatment with M3 or M5 reduced H_2_O_2_-induced ROS production. The present study also showed that SOD and GSH-Px activity markedly decreased in HUVECs and increased the levels of the key cytotoxic lipid peroxide MDA and NO production when they were exposed to 200 *μ*M H_2_O_2_ for 24 h, which was blocked by M3 or M5 cotreatments. Major intracellular antioxidant defenses are the GSH pool and ROS-scavenging enzymes such as GSH-Px and SOD. Increased levels or activation of these endogenous antioxidants or enzymes has been shown to protect cells against oxidative damage. In this study, a marked decrease indeed occurred in the level of GSH after the treatment with H_2_O_2_; as a result, the decreased levels of GSH might be attributed to the less decreased activity of GSH-Px. The imbalance of the H_2_O_2_-induced antioxidant status in HUVECs, such as the changes in the activity of antioxidant enzymes and the depletion of GSH, might be major causes of cell injury. Nevertheless, the cytoprotective effects of M3 or M5 may be mediated, in part, by activation of Nrf2, a redox-regulated transcription factor that binds to the antioxidant response element (ARE). These data suggest that H_2_O_2_-induced ROS production exceeds the natural antioxidant capacity and leads to oxidative stress and that M3 or M5 may exert its protective effect on HUVECs by reducing ROS accumulation and increasing antioxidant enzyme activity.

Finally, in this study, we demonstrated that FWKVV (M3) and FMPLH (M5) could significantly protect cells against H_2_O_2_-induced oxidative stress, DNA damage, and cell apoptosis in cultured HUVECs. Treatment with M3 or M5 reduced the loss of cell viability, improved the antioxidant capacity, and reduced the oxidative damage in HUVECs. Further, we hypothesized that the protective effect of M3 or M5 appears to be mediated by the regulation of the Nrf2 signaling pathway and the maintenance of cellular redox homeostasis. This finding further supports the concept that M3 or M5 may have potential as a vascular protective drug.

## 5. Conclusion

In this work, the protective activity and antioxidation mechanisms of FWKVV (M3) and FMPLH (M5) on HUVECs against H_2_O_2_-caused oxidative injury were researched carefully. When incubated for 24 h, no significant difference was found on the viability of HUVECs between the blank control group and the AP (M1-M10) groups at 100 *μ*M (*P* < 0.05). Moreover, FWKVV (M3) AND FMPLH (M5) could significantly protect HUVECs from H_2_O_2_-induced oxidative damage by enhancing the contents of endogenous antioxidases, lowering the levels of oxidative products, and helping DNA from oxidative injury. These results suggested that FWKVV (M3) and FMPLH (M5) could serve as great potential antioxidants in the health care products. In addition, the molecular mechanism and *in vivo* antioxidant experiments of FWKVV (M3) and FMPLH (M5) will also be carried out in our laboratory.

## Figures and Tables

**Table 1:** Protective capacity of FWKVV (M3) on DNA against H_2_O_2_-induced oxidative damage in HUVECs.

Group	Blank control	Model (H_2_O_2_)	Positive control (NAC)	FWKVV (M3, *μ*M)		
				200	100	50
Cell number (*n*)	104	117	121	113	109	124
HDNA (%)	91.9 ± 4.2	18.2 ± 3.2^**###**^	80.7 ± 3.8^∗∗∗^	69.6 ± 6.9^∗∗∗^	46.8 ± 3.3^∗∗∗^	32.2 ± 2.1^∗∗∗^
TDNA (%)	8.1 ± 4.2	81.8 ± 3.2^**###**^	19.3 ± 4.8^∗^	30.4 ± 6.9^∗∗∗^	53.2 ± 2.5^∗∗∗^	67.8 ± 3.0^∗∗^
CL (pix)	68.3 ± 3.3	77.3 ± 13.5^**#**^	66.2 ± 3.3^∗∗∗^	63.0 ± 3.2^∗∗∗^	66.6 ± 7.3^∗∗∗^	72.0 ± 5.5
TL (pix)	7.3 ± 0.33	59.3 ± 6.6^**###**^	27.2 ± 1.6^∗∗∗^	29.6 ± 3.3^∗∗∗^	33.7 ± 2.5^∗∗∗^	47.7 ± 3.1^∗∗∗^
TM	0.6 ± 0.03	46.4 ± 8.1^**###**^	5.2 ± 0.24^∗∗∗^	10.9 ± 1.2^∗∗∗^	18.0 ± 2.4^∗∗∗^	39.4 ± 2.8^∗∗∗^
OTM	2.3 ± 0.1	24.5 ± 4.3^**###**^	4.7 ± 0.2^∗∗∗^	8.7 ± 1.0^∗∗∗^	12.6 ± 1.7^∗∗∗^	21.1 ± 1.5^∗∗^

All data are presented as the mean ± SD of triplicate results (*n* = 3). ^#^*P* < 0.05 and ^###^*P* < 0.001 vs. the blank control group; ^∗^*P* < 0.05, ^∗∗^*P* < 0.01, and ^∗∗∗^*P* < 0.001 vs. the oxidative damage model group.

**Table 2:** Protective capacity of FMPLH (M5) on DNA against H_2_O_2_-induced oxidative damage in HUVECs.

Group	Blank control	Model (H_2_O_2_)	Positive control (NAC)	FMPLH (M5, *μ*M)		
				200	100	50
Cell number (*n*)	104	117	121	103	114	106
HDNA (%)	91.9 ± 4.2	18.2 ± 3.2^**###**^	80.7 ± 3.8^∗∗∗^	62.3 ± 6.9^∗∗∗^	44.7 ± 5.4^∗∗∗^	31.6 ± 3.8
TDNA (%)	8.1 ± 4.2	81.8 ± 3.2^**###**^	19.3 ± 4.8^∗^	37.7 ± 6.9^∗∗∗^	55.3 ± 5.4^∗∗∗^	68.4 ± 8.3^∗∗^
CL (pix)	68.3 ± 3.3	77.3 ± 13.5^**#**^	66.2 ± 3.3^∗∗∗^	62.0 ± 7.3^∗∗∗^	70.4 ± 6.5^∗^	76 ± 6.2
TL (pix)	7.3 ± 0.33	59.3 ± 6.6^**###**^	27.2 ± 1.6^∗∗∗^	35.4 ± 3.3^∗∗∗^	39.0 ± 2.9^∗∗∗^	51.7 ± 4.6^∗∗^
TM	0.6 ± 0.03	46.4 ± 8.1^**###**^	5.2 ± 0.24^∗∗∗^	16.7 ± 1.6^∗∗∗^	21.6 ± 2.6^∗∗∗^	40.9 ± 2.3^∗∗^
OTM	2.3 ± 0.1	24.5 ± 4.3^**###**^	4.7 ± 0.2^∗∗∗^	11.5 ± 1.1^∗∗∗^	13.1 ± 1.0^∗∗∗^	22.6 ± 1.6^∗^

All data are presented as the mean ± SD of triplicate results (*n* = 3). ^#^*P* < 0.05 and ^###^*P* < 0.001 vs. the blank control group; ^∗^*P* < 0.05, ^∗∗^*P* < 0.01, and ^∗∗∗^*P* < 0.001 vs. the oxidative damage model group.

## Data Availability

The datasets are available from the corresponding author on reasonable request.
